# Identification of miRNAs Involved in Reprogramming Acinar Cells into Insulin Producing Cells

**DOI:** 10.1371/journal.pone.0145116

**Published:** 2015-12-21

**Authors:** Joan Teichenne, Meritxell Morró, Alba Casellas, Veronica Jimenez, Noelia Tellez, Adrien Leger, Fatima Bosch, Eduard Ayuso

**Affiliations:** 1 Center of Animal Biotechnology and Gene Therapy, Universitat Autònoma de Barcelona, Bellaterra, Spain; 2 Department of Biochemistry and Molecular Biology, School of Veterinary Medicine. Universitat Autònoma de Barcelona, Bellaterra, Spain; 3 CIBER de Diabetes y Enfermedades Metabólicas Asociadas (CIBERDEM), Madrid, Spain; 4 Bellvitge Biomedical Research Institute, IDIBELL, L'Hospitalet de Llobregat, Barcelona, Spain; 5 Laboratoire de Thérapie Génique, INSERM UMR1089, University of Nantes and Atlantic Gene Therapies, Nantes, France; Institut d'Investigacions Biomèdiques August Pi i Sunyer, SPAIN

## Abstract

Reprogramming acinar cells into insulin producing cells using adenoviral (Ad)-mediated delivery of *Pdx1*, *Ngn3* and *MafA* (PNM) is an innovative approach for the treatment of diabetes. Here, we aimed to investigate the molecular mechanisms involved in this process and in particular, the role of microRNAs. To this end, we performed a comparative study of acinar-to-β cell reprogramming efficiency in the rat acinar cell line AR42J and its subclone B13 after transduction with Ad-PNM. B13 cells were more efficiently reprogrammed than AR42J cells, which was demonstrated by a strong activation of β cell markers (Ins1, Ins2, IAPP, NeuroD1 and Pax4). miRNome panels were used to analyze differentially expressed miRNAs in acinar cells under four experimental conditions (i) non-transduced AR42J cells, (ii) non-transduced B13 cells, (iii) B13 cells transduced with Ad-GFP vectors and (iv) B13 cells transduced with Ad-PNM vectors. A total of 59 miRNAs were found to be differentially expressed between non-transduced AR42J and B13 cells. Specifically, the miR-200 family was completely repressed in B13 cells, suggesting that these cells exist in a less differentiated state than AR42J cells and as a consequence they present a greater plasticity. Adenoviral transduction *per se* induced dedifferentiation of acinar cells and 11 miRNAs were putatively involved in this process, whereas 8 miRNAs were found to be associated with PNM expression. Of note, Ad-PNM reprogrammed B13 cells presented the same levels of miR-137-3p, miR-135a-5p, miR-204-5p and miR-210-3p of those detected in islets, highlighting their role in the process. In conclusion, this study led to the identification of miRNAs that might be of compelling importance to improve acinar-to-β cell conversion for the future treatment of diabetes.

## Introduction

Type 1 diabetes (T1D) results from autoimmune destruction of β cells, the insulin-producing cells in the pancreatic islets of Langerhans. According to the International Diabetes Federation, it is estimated that 8.3% of adults (382 million people) have diabetes, and approximately 10% of them are type 1 diabetic patients [1]. Current treatments for T1D include either the administration of exogenous insulin or islet transplantation. However, insulin replacement therapy fails to achieve tight glycemic control, leading to significant morbidity and mortality. Therapeutic benefit has been obtained with islet transplantation, but the scarcity of cadaveric donors and the complications associated with long-term immunosuppression have hampered its broad clinical application. Therefore, the search for alternative sources of insulin-producing cells is of compelling importance for the treatment of T1D.

The conversion of non-β cells into insulin-producing cells *in vivo* is an innovative approach to treat diabetes and circumvents the need for immunosuppression associated with allogeneic transplantation. *In vivo* reprogramming of hepatic cells into insulin-producing cells has been achieved by adenoviral (Ad)-mediated gene transfer of the transcription factors (TFs) *Pdx1*, *Ngn3* and *MafA* (PNM), either individually or in combination [2–7]. Although reprogrammed hepatocytes or ductal cells in the liver were able to secrete insulin and ameliorate hyperglycemia, full conversion into *bona-fide* (mature) β cells was not achieved. On the contrary, *in vivo* ectopic expression of PNM in adult acinar cells via Ad vectors converted transduced cells into insulin-producing cells that closely resembled islet β cells [8,9]. Notably, reprogrammed exocrine cells aggregated into islet-like clusters and mediated long-term remission of diabetes [8].

AR42J is a rat cell line that was originally derived from a chemically induced pancreatic acinar carcinoma [10]. This cell line has been described as amphicrine because it does not only possesses exocrine properties, such as the synthesis, storage and secretion of digestive enzymes, but also displays several neuroendocrine properties, including an electrically excitable membrane [11]. The B13 subclone was isolated in 1996 from the AR42J cell line, and it presented a much greater β cell reprogramming efficiency than its parental cell line after treatment with hepatocyte growth factor and activin A [12]. Since then, B13 cells have been widely used as a model system to study the molecular mechanisms that mediate acinar-to-β cell reprogramming under several experimental conditions, including culture with growth factors, Ad-mediated overexpression of different combinations of TFs or treatment with protein-transduction-domain containing TFs [13–18]. Indeed, *in vitro* transduction of B13 cells with Ad-PNM reprogrammed said cells into insulin-producing cells, which were able to relieve diabetes upon transplantation into NOD-SCID mice [13].

Nonetheless, the ability to convert AR42J (parental) cells into β cells by forced overexpression of TFs using Ad vectors has never been evaluated.

The development of insulin-producing cells in the pancreas is not only controlled by TFs but also by microRNAs (miRNAs) [19]. Mature miRNAs are short (~22 bp), non-coding RNAs that can negatively or positively regulate gene expression at the post-transcriptional level by inhibiting translation, causing mRNA degradation or inhibiting the production of long non-coding RNAs that are complementary to select genes [20]. Like TFs, miRNAs have also been used to mediate the direct reprogramming of one cell type into another, both *in vitro* and *in vivo*, and have in some cases elicited greater reprogramming efficiencies than standard methods using TFs [21]. Regarding β cell reprogramming, performing a co-transfection of miRNA-302 and PNM has been recently reported to improve the reprogramming efficiency of cells from a human hepatocyte cell line into pancreatic progenitor cells [22], and miRNA-375 was reported to promote β pancreatic differentiation in human induced pluripotent stem (hiPS) cells [23].

The goal of the present study was to gain further insight into the mechanisms underlying the reprogramming of exocrine cells towards a β-cell phenotype, and particularly, to identify novel miRNAs involved in said process. To this end, we performed a comparative study in the rat acinar cell line AR42J and B13 cells after transduction with Ad vectors that were co-expressing PNM. We found that B13 cells were more efficiently differentiated into insulin-producing cells than the parental AR42J cells. Using miRNome-screening panels we identified 69 miRNAs that are putatively involved in the reprogramming process. We observed that 59 of them were differentially expressed between non-transduced AR42J and B13 cells, 11 miRNAs were modified by adenovirus transduction, whereas 8 miRNAs were found to be associated with PNM expression. A selection of these candidates was further analyzed by individual qPCR analysis using rat islets and exocrine tissue as controls and >80% of the selected miRNAs showed the same expression pattern observed in the preliminary screening. Interestingly, PNM overexpression in B13 cells upregulated the expression of miR-137-3p, miR-135a-5p and miR-204-5p and repressed the expression of miR-210-3p to the same levels of those detected in rat islets, highlighting the potential role of these miRNAs in the reprogramming process. Altogether, this study identified candidate miRNAs that might be used to improve acinar-to-β cell conversion for the future treatment of diabetes.

## Materials and Methods

### Cell culture and adenoviral transduction

The parental rat acinar cell line AR42J was obtained from ATCC (CRL-1492). The subclone AR42J-B13 (B13) was a kind gift from Dr. Matthew Wright (Newcastle University, UK) [24]. Both cell lines were grown in DMEM (1 g/l) (Gibco, Carlsbad, CA, USA), supplied with L-glutamine (Gibco), pyruvate (Gibco), 10% FBS (Gibco) and penicillin/streptomycin (complete media). For transduction experiments, cells were plated 3 days prior to transduction in 12-well plates. Before transduction, complete media was replaced with DMEM supplemented with 2% FBS (transduction media). AR42J and B13 cells were independently transduced with a dose of 2000 vector particles (vp)/cell, diluted in 50 μl of PBS Ca/Mg (PAA, Pasching, Austria). The transduction media was replaced with complete media at 16 hours post-transduction.

### Islets and exocrine fraction isolation

Experimental procedures were reviewed and approved by the Ethical Committee of the University of Barcelona. Pancreatic islets and islet-depleted exocrine fractions were obtained from 8 weeks old male Wistar rats (Janvier, Saint Berthevin, France) by collagenase digestion (Collagenase P; Boehringer Mannheim Biochemicals, Mannheim, Germany) as previously performed [25]. Briefly, following density gradient (Histopaque ^®^ -1077, Sigma Immunochemicals, St Louis, MO, USA), the interphase (islets) and pellet (exocrine cells) fractions were separately collected and washed three times with M199 medium (Sigma). Pancreatic islets were hand-picked under a stereomicroscope two or three times, until pure preparations were obtained. Similarly, exocrine fractions were hand-picked under the stereomicroscope to remove any remaining islets.

### Adenoviral stocks

First-generation recombinant adenoviral vectors were used in these experiments. Adenoviral vectors encoding a polycistronic construct expressing *GFP*, *Pdx1*, *Ngn3* and *MafA* under the control of the chicken β-actin (CAG) ubiquitous promoter (Ad-PNM) [3] were kindly provided by Dr. Jonathan Slack (University of Minnesota, MN, USA). Both adenoviral vectors encoding GFP under the control of the cytomegalovirus (CMV) promoter (Ad-GFP) and null adenoviral vectors (Ad-null), which retained equal infectivity but did not carry any transgene, were produced at the Vector Production Unit at the Center of Animal Biotechnology and Gene Therapy at Universitat Autònoma de Barcelona (Bellaterra, Spain) as previously described [26].

### mRNA expression analysis

AR42J and B13 cells were cultured in 12-well plates and harvested 4 days post-transduction in 800 μl TriPure Isolation Reagent (Roche, Basel, Switzerland), and total RNA was isolated using an RNeasy Kit (Qiagen, Hilden, Germany). Total RNA was extracted from 100–200 isolated islets and 35 mg of exocrine fractions using 1 ml Tripure Isolation Reagent (Roche) and Rneasy Mini Kit (Qiagen). cDNA was synthesized from 1 μg total RNA by reverse transcription using a Transcriptor First Strand cDNA Synthesis Kit (Roche). Quantitative real-time PCR (qPCR) was performed using SYBR Green I Master (Roche) in a LightCycler^®^ 480 II (Roche) with specific primers (0.2 μM) (S1 Table). Ct values higher than 40 were excluded from analysis. Data were analyzed using the 2^ΔΔCt^ method [27] and normalized to *Rplp0* expression.

### Immunostaining

B13 cells were plated 3 days before adenoviral transduction in 24-well plates containing round coverslips (Marienfield, Lauda-Königshofen, Germany). Culture plates were treated with 0.1% gelatin for 1 h at 37°C before plating the cells. Four days post-transduction, the cells were fixed with a formaldehyde solution (2% formaldehyde in PBS), washed twice with PBS and kept in wash buffer (0.1% BSA in PBS). For fluorescent insulin immunostaining, the cells were treated with blocking buffer (5% horse serum and 0.2% Triton in PBS) for 45 minutes at room temperature. Following this, the cells were incubated for 1 h at room temperature with a primary antibody (Guinea Pig Anti-insulin; Sigma-Aldrich I-8510, St. Louis, MO, USA) that was diluted 1:500 in dilution buffer (1% BSA, 1% horse serum, 0.2% Triton in PBS) and then incubated with secondary antibody (1:300; Alexa Fluor^®^ 568 Goat anti-Guinea Pig IgG; Molecular Probes A-11075, Carlsbad, CA, USA) and Hoescht (1:100; Sigma-Aldrich). The coverslips were subsequently removed and mounted on microscope slides. The slides were observed under a microscope (Eclipse E800; Nikon, Tokyo, Japan) that was connected to a video camera and an image analyzer (NIS-ELEMENTS AR 2.30; Imaging Software Nikon, Tokyo, Japan).

### Insulin content

B13 cells were plated 3 days before transduction in 6-well plates. The cells were transduced with a dose of 2000 vp of Ad-PNM per cell, diluted in 150 μl PBS Ca/Mg. Three days post-transduction, the cells were harvested in 400 μl RIPA buffer (50 mM Tris-HCl pH 8, 150 mM NaCl, 1% NP-40, 0.5% Na-deoxycholate, 5 mM EDTA, 0.1% SDS, 2 mM Na_3_VO_4_, 10 mM NaF, 10 mM Na_4_P_2_O_7_) containing orthovanadate and protease inhibitors. Cell extracts were sonicated, and insulin content was quantified using a rat insulin RIA Kit (Millipore, Billerica, MA, USA). Protein concentration was determined using a BCA kit (Pierce, Rockford, IL, USA) to normalize insulin levels against total protein content.

### Western blot analysis

For total protein extracts, cells were homogenized in RIPA lysis buffer containing orthovanadate and protease inhibitors. A total of 20 μg of protein was loaded per sample on 10% SDS-PAGE gels. Proteins were transferred onto Immobilon-P membranes (Millipore). The membranes were probed against E-cadherin (H-108, sc-7870, Santa Cruz Biotechnology, Inc) and α-Tubulin (Abcam ab4074, Cambridge, U.K.). Detection was performed using the corresponding horseradish peroxidase-labelled secondary antibodies and Western blotting detection reagent (ECL Plus; Amersham, Freiburg, Germany).

### miRNome panels

AR42J and B13 cells were plated 3 days prior to transduction in 12-well plates. The cells were harvested 4 days post-transduction in 350 μl lysis solution containing 10 μl/ml β-mercaptoethanol and 1 μl RNA isolation spike-in mix (UniSp2, UniSp4, UniSp5), and total RNA was isolated using a miRCURY^™^ Isolation Kit—Cell & Plant (Exiqon, Vedbaek, Denmark). First-strand cDNA synthesis was performed using 40 ng of template total RNA together with 2 μl cDNA synthesis RNA spike-in mix (cel-miR-39-3p and UniSp6) with a Universal cDNA synthesis kit II (Exiqon). To analyze miRNA profiles, Mouse&Rat miRNome Panels I+II V3 (Exiqon), which contained 752 unique primer sets, were used according to the manufacturer’s protocol. qPCR was performed using ExILENT SYBR^®^ Green master mix (Exiqon) in a LightCycler^®^ 480 II (Roche). For data analysis, Ct values were normalized by a calibration factor that was obtained from interplate calibrators. Raw data from miRNome Panels is available in S2 Table. RQ values were obtained using the 2^ΔΔCt^ method [27] and normalized by the RQ value of miR-16-5p, which led to the best stability value according to NormFinder Software [28]. Principal component analysis and heatmap generation were performed with an open source R script (available at http://a-slide.github.io/MirStat/) together with detailed online documentation. Prior to analysis, qPCR data were manually preprocessed. To minimize stochasticity observed at high Ct, values above 35 were considered non-detected (ND). Additionally, miRNAs with an intra-replicate Ct fold-change above 2 were excluded from analysis. In the lists of the most differentially expressed miRNAs, 3 detected values versus ≥ 2 ND values were required to receive the label “Detected *vs*. ND”. Ct values corresponding to the miRNAs shown in Tables 1, 2 and 3 are available in S3, S4 and S5 Tables, respectively.

**Table 1:** Differentially expressed miRNAs comparing AR42J cells to B13 cells. To minimize stochasticity observed at high Ct, values above 35 were considered non-detected (ND). Additionally, miRNAs with an intra-replicate Ct fold-change above 2 were excluded from analysis. 3 detected values versus ≥ 2 ND values were required to receive the label “Detected *vs*. ND”. P values were determined by using Student’s *t* test. n = 3 wells per group.

"AR42J no Ad" vs "B13 no Ad"			"B13 no Ad" vs "AR42J no Ad"		
microRNA	Ratio	P value	microRNA	Ratio	P value
miR-200c-3p	Detected vs N.D.	-	miR-325-3p	Detected vs N.D.	-
miR-141-3p	Detected vs N.D.	-	miR-325-5p	Detected vs N.D.	-
miR-141-5p	Detected vs N.D.	-	miR-92b-3p	Detected vs N.D.	-
miR-378a-3p	Detected vs N.D.	-	miR-142-3p	Detected vs N.D.	-
miR-205-5p	Detected vs N.D.	-	miR-137-3p	Detected vs N.D.	-
miR-149-5p	Detected vs N.D.	-	miR-27a-5p	Detected vs N.D.	-
miR-28-5p	Detected vs N.D.	-	miR-330-3p	6.8	0.0039
miR-200b-3p	Detected vs N.D.	-	miR-101a-3p	3.75	< 0.0001
miR-181b-5p	Detected vs N.D.	-	miR-301a-5p	3.55	0.0008
miR-200c-5p	Detected vs N.D.	-	miR-328a-3p	3.52	0.0082
miR-429	Detected vs N.D.	-	miR-301a-3p	3.31	< 0.0001
miR-200a-3p	Detected vs N.D.	-	miR-455-3p	3.07	0.0183
miR-483-3p	21.84	0.0004	miR-31a-3p	2.81	0.0003
miR-28-3p	8.48	0.0129	miR-193-5p	2.57	0.0339
miR-34a-5p	4.8	< 0.0001	miR-2137	2.57	0.0012
let-7i-5p	4.57	0.0007	miR-1839-5p	2.55	0.0246
miR-181a-5p	4.21	0.0002	miR-106b-5p	2.53	< 0.0001
let-7i-3p	4.17	0.0004	miR-7a-5p	2.3	0.038
miR-192-5p	3.09	0.0004	miR-153-3p	2.25	0.0016
miR-30a-5p	2.89	0.0006	miR-365-3p	2.19	0.0033
miR-194-5p	2.86	0.0004	miR-25-3p	2.15	0.0003
miR-347	2.79	0.0152	miR-29a-5p	2.09	0.0055
miR-122-5p	2.69	0.0012	miR-31a-5p	2.07	0.0012
miR-374-5p	2.54	0.0038			
miR-30a-3p	2.47	0.0016			
let-7b-5p	2.42	0.0041			
let-7g-5p	2.34	0.0005			
miR-361-5p	2.32	0.0103			
miR-204-5p	2.32	0.0038			
miR-497-5p	2.22	0.0015			
let-7d-5p	2.18	0.0078			
miR-500-3p	2.13	0.0574			
miR-125b-5p	2.08	0.0001			
miR-181d-5p	2.06	0.0105			
miR-26b-5p	2.03	0.0007			
miR-421-3p	2.02	0.0511			

**Table 2:** Differentially expressed miRNAs comparing B13 cells transduced with Ad-GFP to not transduced B13 cells. To minimize stochasticity observed at high Ct, values above 35 were considered non-detected (ND). Additionally, miRNAs with an intra-replicate Ct fold-change above 2 were excluded from analysis. 3 detected values versus ≥ 2 ND values were required to receive the label “Detected *vs*. ND”. P values were determined by using Student’s *t* test. n = 3 wells per group.

"B13 Ad-GFP" vs "B13 no Ad"			"B13 no Ad" vs "B13 Ad-GFP"		
microRNA	Ratio	P value	microRNA	Ratio	P value
miR-2137	9.1	0.0007	miR-210-3p	8.71	< 0.0001
miR-335-3p	3.23	0.0001	miR-181a-5p	2.65	0.026
miR-148a-5p	2.74	0.0151	miR-483-3p	2.36	0.003
miR-421-3p	2.5	0.002	miR-181c-5p	2.1	0.0075
miR-350	2.48	0.0066			
miR-204-5p	2.23	0.0041			
miR-132-3p	2.17	0.0088			

**Table 3:** Differentially expressed miRNAs comparing B13 cells transduced with Ad-PNM to B13 cells transduced with Ad-GFP. To minimize stochasticity observed at high Ct, values above 35 were considered non-detected (ND). Additionally, miRNAs with an intra-replicate Ct fold-change above 2 were excluded from analysis. 3 detected values versus ≥ 2 ND values were required to receive the label “Detected *vs*. ND”. P values were determined by using Student’s *t* test. n = 3 wells per group.

"B13 Ad-PNM" vs "B13 Ad-GFP"			"B13 Ad-GFP" vs "B13 Ad-PNM"		
microRNA	Ratio	P value	microRNA	Ratio	P value
miR-134-5p	Detected vs N.D.	-	miR-335-3p	3.26	0.0006
miR-455-3p	3.78	0.0021	miR-148a-5p	2.24	0.038
miR-384-5p	3.17	0.0198			
miR-137-3p	2.88	0.0333			
miR-135a-5p	2.85	0.0126			
miR-22-5p	2.09	0.0892			

### Individual miRNA qPCR assays

Total RNA from 7 mg of exocrine fraction and 100–200 islets was isolated in 600 μl lysis solution containing 10 μl/ml β-mercaptoethanol and 1 μl RNA isolation spike-in mix using a miRCURY^™^ Isolation Kit—Cell & Plant (Exiqon). RNA from AR42J and B13 cells was isolated as described above. First-strand cDNA synthesis was performed using 10 ng of total RNA per sample with a Universal cDNA synthesis kit II (Exiqon). qPCR was performed using ExILENT SYBR^®^ Green master mix (Exiqon) in a LightCycler^®^ 480 II (Roche) with specific primers provided by Exiqon according to the manufacturer’s protocol (microRNA LNA^™^ PCR primer sets). Ct values higher than 35 were considered not detected. Data were analyzed using the 2^ΔΔCt^ method [27] and normalized by miR-16-5p expression values.

### Statistical analysis

All values are expressed as the mean ± SEM. Significant differences were tested according to a Gaussian distribution of the samples by Student’s t test; for multiple comparisons, one-way ANOVA followed by post hoc Dunnett’s test or Tukey’s test were used, with a discriminating *p* value of 0.05.

## Results

### B13 cells were more efficiently reprogrammed into a β-cell-like phenotype than AR42J cells

**Fig 1:**
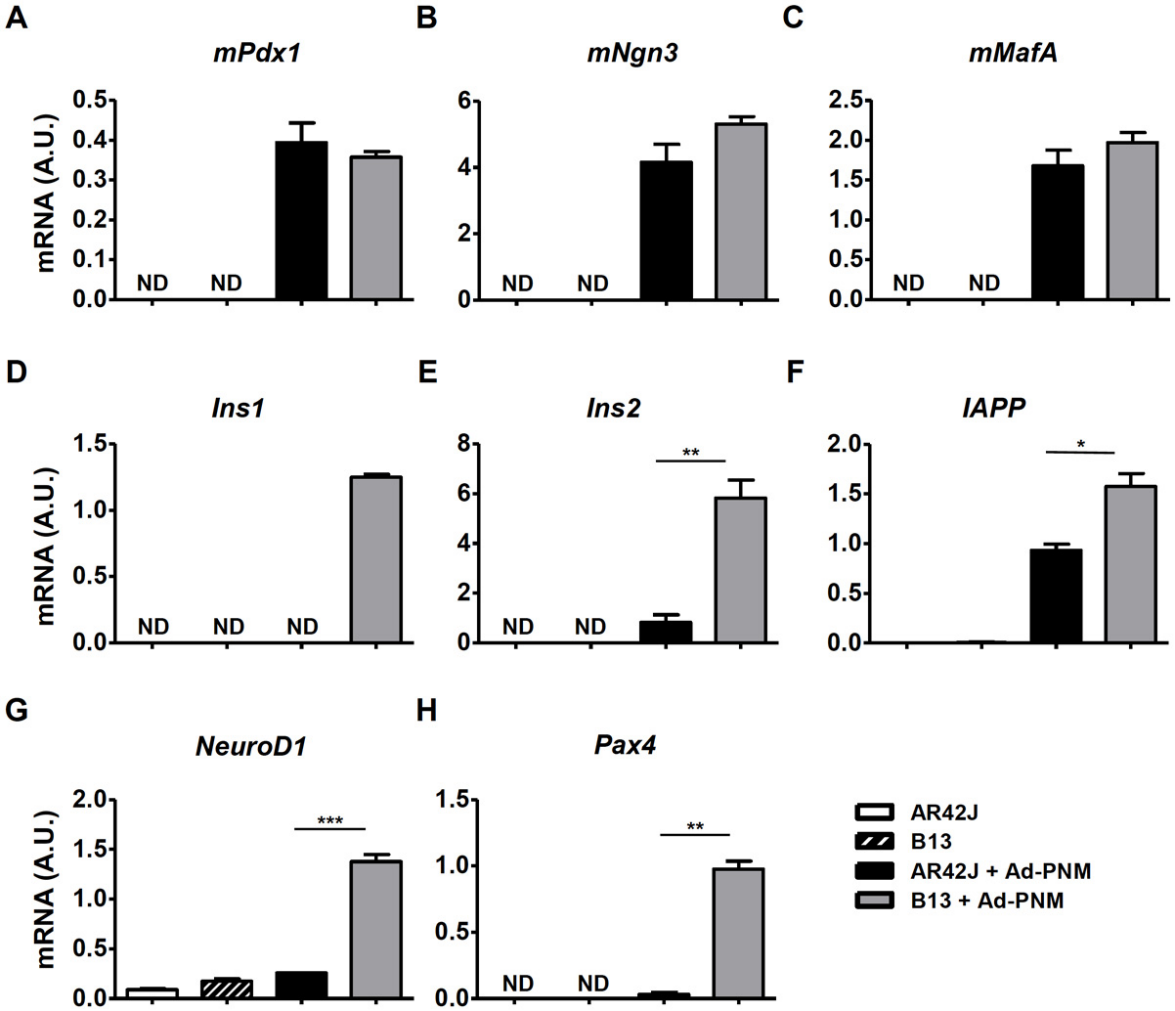
Expression levels of β cell markers after Ad-PNM transduction in AR42J and B13 cells. Relative mRNA expression levels of mouse *Pdx1* (**A**), *Ngn3* (**B**) and *MafA* (**C**) and rat endocrine markers *Ins1* (**D**), *Ins2* (**E**), *IAPP* (**F**), *NeuroD1* (**G**) and *Pax4* (**H**) in AR42J and its subclone B13 at 4 days post-transduction. The results are depicted as means ± SEM. ND, not detected. n = 3 wells per group. *p < 0.05, **p < 0.01, ***p < 0.01, as determined by using Student’s *t* test to compare AR42J cells transduced with Ad-PNM to B13 transduced with Ad-PNM. A.U., arbitrary units.

To compare their efficiencies of reprogramming into insulin-producing cells, AR42J and B13 cells were transduced with adenoviral vectors bearing a polycistronic expression cassette that encoded the murine transcription factors *Pdx1 (mPdx1)*, *Ngn3* (*mNgn3)* and *MafA (mMafA)*, as well as a *GFP* marker gene, all of which were under the control of a CAG promoter (Ad-PNM) [3]. Both AR42J and B13 cells were efficiently transduced by Ad-PNM, as indicated by high expression levels of *mPdx1*, *mNgn3* and *mMafA* (Fig 1A–1C) and high GFP protein production in both cell lines (S1 Fig). The adenoviral-mediated ectopic expression of *mPdx1*, *mNgn3* and *mMafA* induced expression of endocrine genes such as *Ins2*, *IAPP*, *NeuroD1*, and *Pax4* in both AR42J and B13 cells, although much higher expression levels (ranging from 1.5-fold to 30-fold) were found in B13 cells (Fig 1E–1H). Moreover, transduction with Ad-PNM could not activate *Ins1* expression in the parental cell line AR42J (Fig 1D). The enhanced reprogramming efficiency of B13 cells was not due to differential expression levels of *mPdx1*, *mNgn3* or *mMafA* between AR42J and B13 cells (Fig 1A–1C); rather, it was likely a result of intrinsic characteristics of the B13 subclone.

**Fig 2:**
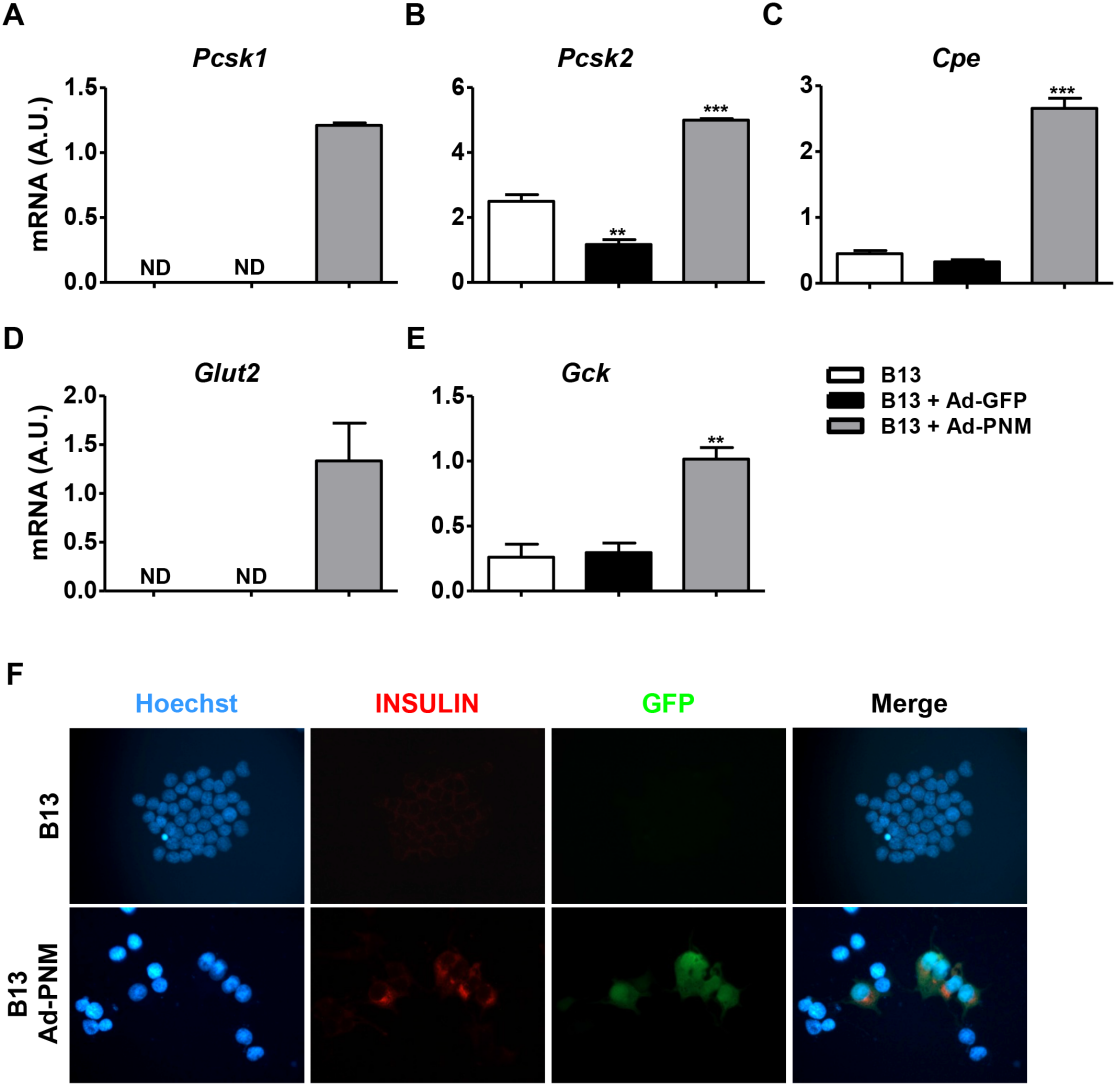
Expression levels of the insulin processing enzymes Glut 2 and Gck and insulin protein production in reprogrammed B13 cells. Relative mRNA expression levels of the insulin processing enzymes *Pcsk1* (**A**), *Pcsk2* (**B**) and *Cpe* (**C**), the glucose transporter *Glut2* (**D**) and glucokinase (*Gck*) (**E**). (**F**) Representative images of immunocytochemical detection of insulin (in red) and GFP (in green) protein production in reprogrammed B13 cells. Blue, Hoescht (nuclei). All of the analyses were performed at 4 days after transduction with Ad-PNM or Ad-GFP vectors. The results are depicted as means ± SEM. ND, not detected. n = 3 wells per group. **p<0.01, ***p<0.001, as determined by one-way ANOVA followed by a post hoc Dunnett’s post test. A.U., arbitrary units.

Ad-PNM reprogrammed B13 cells also exhibited increased expression of the pro-insulin processing proteins (convertases) *Pcsk1*, *Pcsk2* and *Cpe* (Fig 2A–2C). Accordingly, insulin proteins were detected by immunostaining in the cytoplasms of B13 cells that were transduced with Ad-PNM, which also expressed GFP (Fig 2F). Quantifying the intracellular insulin content by radioimmunoassay further confirmed the existence of insulin production in the Ad-PNM B13 cells (0.89±0.07 ng/mg total protein in Ad-PNM transduced B13 cells *vs* 0.13±0.003 ng/mg in non-transduced B13 cells). Although expression of both the glucose transporter *Glut2* and the glucose phosphorylating enzyme glucokinase (*Gck*), the glucose sensor molecules of mature β cells, was induced in Ad-PNM reprogrammed B13 cells (Fig 2D and 2E), they did not show glucose responsiveness (data not shown), which is in accordance with what has been previously described [12,13,15,16]. In this regard, the mRNA expression levels of all the endocrine markers analyzed was significantly lower in reprogrammed B13 cells compared with primary rat endocrine cells (isolated pancreatic islets) (S2 Fig), which further supports the hypothesis that Ad-PNM B13 cells were not fully differentiated.

**Fig 3:**
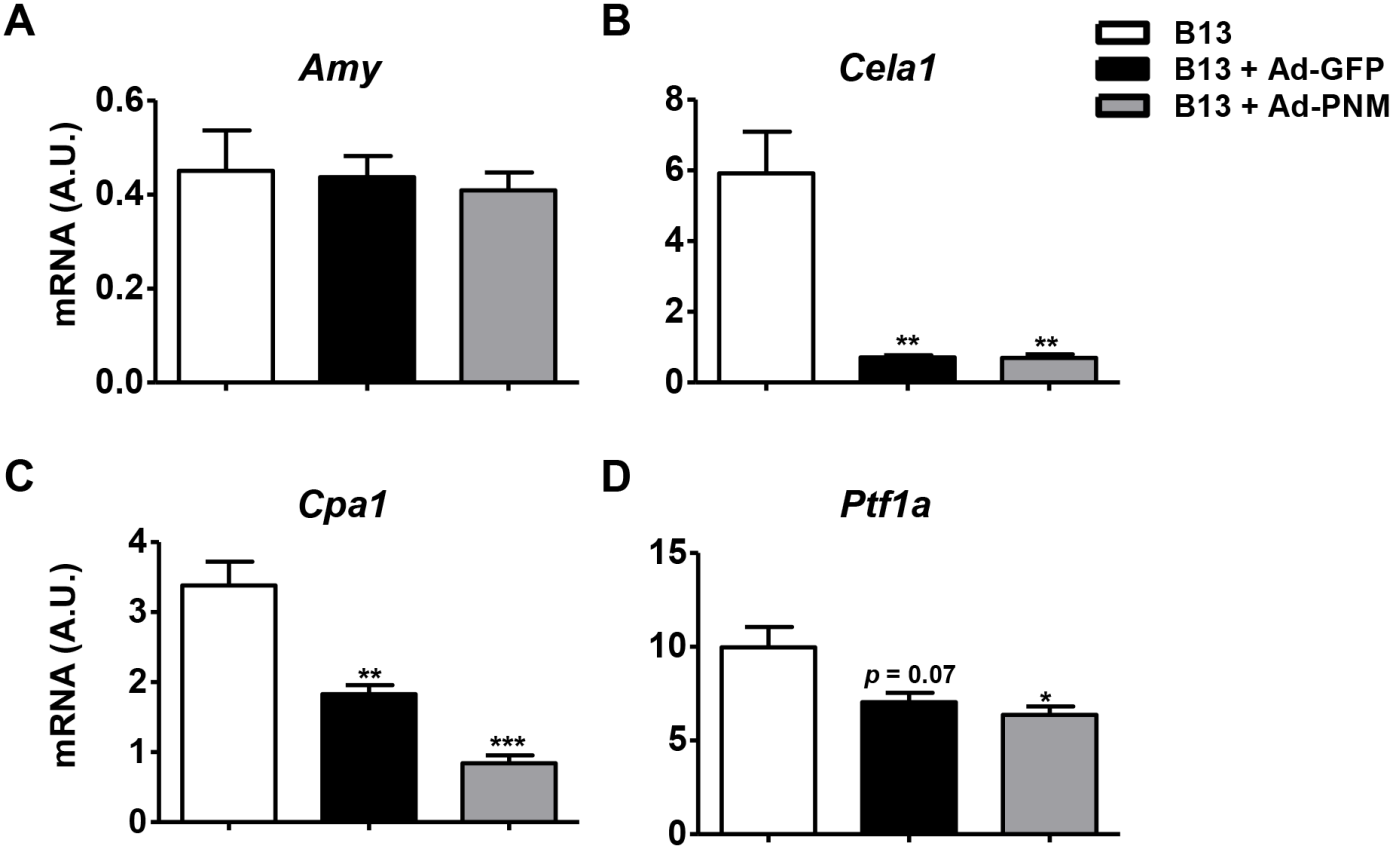
Downregulation of exocrine markers after adenoviral transduction. Relative mRNA expression levels of the exocrine markers *Amy* (**A**), *Cela1* (**B**), *Cpa1* (**C**) and *Ptf1a* (**D**) at 4 days post-transduction with Ad-PNM or Ad-GFP vectors. The results are depicted as means ± SEM. n = 3 wells per group. *p< 0.05, **p<0.01, ***p<0.001, as determined by one-way ANOVA followed by a post hoc Dunnett’s post test. A.U., arbitrary units.

In agreement with previous reports [13,15], transduction with Ad-PNM elicited β-cell-like specific reprogramming, which was indicated by an inability to detect the expression of non-β cell endocrine markers such as glucagon in reprogrammed B13 cells (data not shown). Moreover, Ad-PNM B13 cells down-regulated the expression of the exocrine markers *Cela1*, *Cpa1*, *Ptf1a* (Fig 3B–3D); however, intriguingly, expression levels of amylase (*Amy*) were unchanged (Fig 3A). The four aforementioned exocrine markers were significantly much less expressed in non-transduced B13 cells compared with rat primary exocrine tissue and the parental cell line AR42J (S3 Fig), suggesting basal partial dedifferentiation of B13 cells that likely contributes to the plasticity of said cells to be reprogrammed toward an endocrine phenotype.

### Adenoviral transduction down-regulated exocrine markers in B13 cells

Adenoviral transduction itself has been reported to play a role in reprogramming cells to have an endocrine phenotype in certain cell types [7,29]. The transduction of B13 cells with adenoviral vectors that encoded only the GFP marker gene (Ad-GFP) did not induce the expression of any of the β cell markers that were analyzed (*Ins2*, *IAPP*, *NeuroD1*, *Pax4*, *PcsK1*, *Pcsk2*, *Cpe*, *Glut2* and *Gck*) (Fig 2A–2E and data not shown). However, similarly to the effect mediated by Ad-PNM transduction, decreased expression levels of the exocrine markers *Cela1*, *Cpa1* and *Ptf1a* were observed upon transfection of B13 cells with either Ad-GFP (Fig 3B–3D) or null adenoviral vectors (Ad-null) (S4 Fig). Thus, adenoviral transduction, independently of the encoded transgene, mediated acinar cell dedifferentiation, which may facilitate the reprogramming of B13 cells toward an insulin-producing phenotype.

### Identification of miRNAs involved in the reprogramming of B13 cells toward insulin producing cells

To explore the impact of the non-coding transcriptome in the reprogramming process, the expression levels of 752 separate rodent miRNAs under four different experimental conditions were screened: (i) non-transduced AR42J cells, (ii) non-transduced B13 cells, (iii) B13 cells transduced with Ad-GFP vectors and (iv) B13 cells transduced with Ad-PNM vectors.

**Fig 4:**
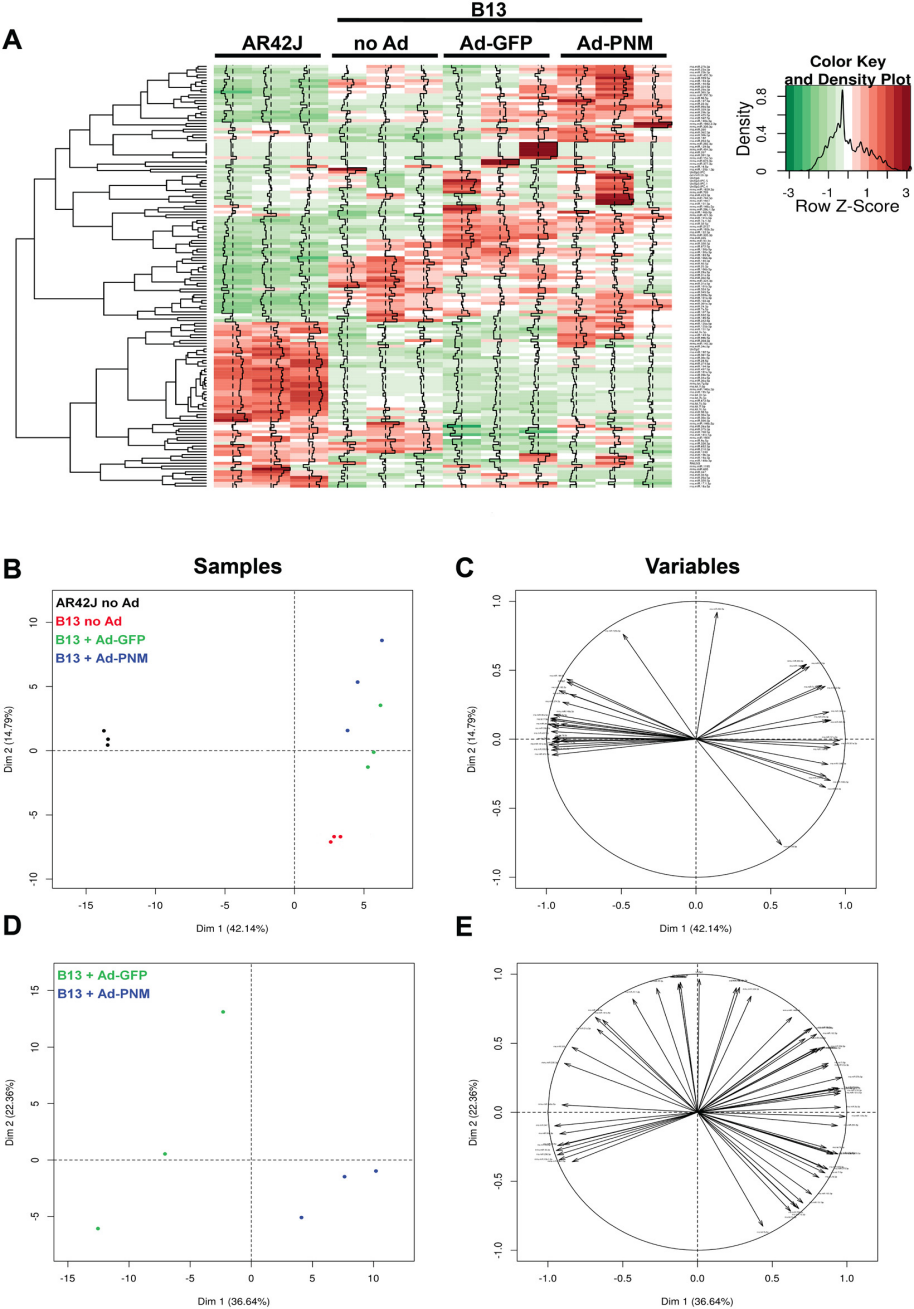
miRNA expression profiles of AR42J and B13 cells that were either transduced or not transduced with adenoviral vectors. (**A**) Heatmap for all valid miRNAs detected in samples, calculated from pairwise Pearson distances. Green: lower Ct, Red: higher Ct (**B-C**). First plan of a principal component analysis (PCA) from data reduced, centered and classified by hierarchical cluster analysis. (**B**) and (**C**) represent data obtained from a projection of all samples, and (**D**) and (**E**) show only data from B13 cells that were transduced with either Ad-GFP or Ad-PNM. (**B**) and (**D**) represent a projection in individual space (samples), whereas (**C**) and (**E**) represent a projection in variable space (miRNAs).

Although the miRNome data were highly reproducible among biological replicates, completely different patterns of miRNA expression were obtained in all four experimental conditions, with AR42J and non-transduced B13 cells showing the highest variability, as depicted in a heatmap that compared all of the groups of samples (Fig 4A). These results suggest that the miRNome of B13 cells could be directly associated with the reprogramming capacity of these cells.

A Principal Component Analysis (PCA) further confirmed that very different miRNA profiles existed between the parental cell line AR42J and its subclone B13, as AR42J and B13 (non-transduced and adenoviral-transduced) cells were clearly opposed on the first principal component (Fig 4B and 4C). Moreover, B13 cells that were transduced with Ad vectors were separated from non-transduced B13 cells on the second component, indicating that adenoviral transduction was responsible for the differential expression of a limited group of miRNAs, which in turn were not common to those that exhibited differences in the miRNA profiles of non-transduced cells (Fig 4B and 4C). Conversely, Ad-PNM- and Ad-GFP-transduced B13 cells clustered after performing PCA on all four experimental groups (Fig 4B and 4C), which was probably because the tremendous number of differentially expressed miRNAs between AR42J and B13 miRNA profiles masked the reduced number of differentially expressed miRNAs between these two groups. Nevertheless, when PCA was performed on these two groups alone, they were separated on the first component (Fig 4D and 4E), confirming that ectopic overexpression of *mPdx1*, *mNgn3* and *mMafA* also accounted for differences in their miRNA profiles.

**Fig 5:**
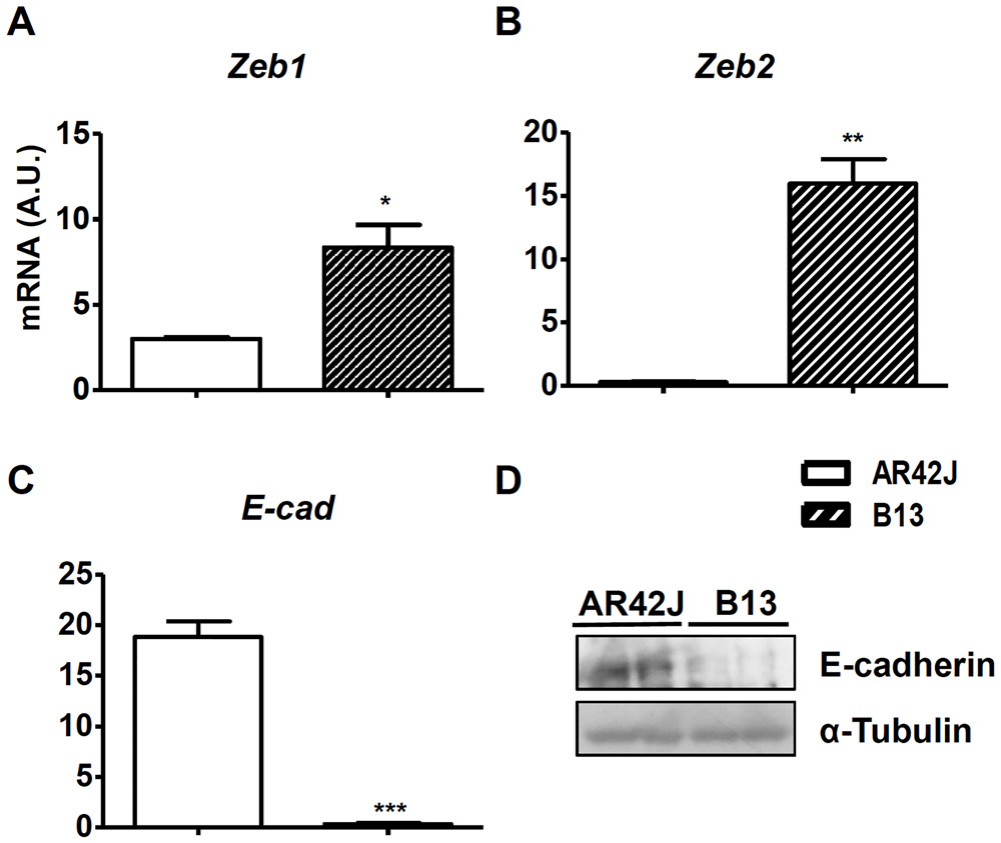
Expression levels of endothelial and mesenchymal markers in AR42J and B13 cells. Relative mRNA expression levels of the mesenchymal factors *Zeb1* (**A**) and *Zeb2* (**B**) and the endothelial marker *E-cad* (**C**) in AR42J and B13 cells. E-cadherin protein levels in AR42J and B13 cells (**D**). The results are depicted as means ± SEM. n = 3 wells per group. *p < 0.05, **p < 0.01, ***p < 0.01, as determined by using Student’s *t* test to compare AR42J cells to B13 cells. A.U., arbitrary units.

To identify the most differentially expressed miRNAs, candidates with expression levels that were at least two-fold different between experimental conditions, as well as miRNAs that were exclusively expressed in only one group, were extracted. A total of 59 miRNAs were found to be differentially expressed between non-transduced AR42J and B13 cells (Table 1). Of these, 24 and 17 miRNAs were up-regulated and down-regulated, respectively, in AR42J *vs*. B13 cells; furthermore, 12 were only detected in AR42J cells and 6 showed exclusive expression in B13 cells (Table 1). Notably, all members of the miR-200 family (miR-200a, miR-200b, miR-200c, miR-141-3p, miR-141-5p and miR-429), which has been shown to be enriched in differentiated tissues such as ectoderm and endoderm and is largely excluded from the mesoderm [30–32], were specifically expressed in AR42J cells and absent in the B13 subclone. miR-200 family members target and inhibit ZEB1 and ZEB2 factors which are key factors in epithelial-to-mesenchymal transition (EMT) [33–35]. Also, overexpression of miR-200c leads to increased expression of E-cadherin, which maintains epithelial phenotypes in cells [33]. The expression levels of *Zeb1* and *Zeb2* were significantly higher in the B13 subclone compared to AR42J cells (2.7- and 57-fold, respectively) (Fig 5A and 5B), whereas expression of *E-cad* was 52-fold lower (Fig 5C), which agree with the expected biological activity of miR-200 family in each cell type. Accordingly, E-Cadherin was detected in AR42J cells at the protein level but undetectable in B13 cells (Fig 5D).

Although Ad-transduced B13 cells were separated from non-transduced B13 cells on the second component in the PCA analysis (Fig 4B and 4C), Ad-GFP vectors only induced up-regulation of 7 miRNAs and down-regulation of 4 miRNAs in comparison with non-transduced B13 cells (Table 2).

miRNA profiles that corresponded to Ad-PNM- and Ad-GFP-transduced B13 cells were compared to identify differentially expressed miRNAs due to ectopic expression of PNM (Table 3). B13 cells that were transduced with Ad-PNM showed upregulation of 6 miRNAs (miR-134-5p, miR-455-3p, miR-384-5p, miR-137-3p, miR-135a-5p and miR-22-5p) and downregulation of 2 miRNAs (miR-335-3p and miR-148a-5p).

In conclusion, we identified 69 miRNAs putatively involved in the reprogramming process of acinar cells towards insulin producing cells using miRNome panels. 9 miRNAs were present in two categories; 5 of them were common between Tables 1 and 2 (miR-181a-5p, miR-204-5p and miR-2137, miR-421-3p and miR-483-3p), two miRNAs were present in both Tables 2 and 3 (miR-148a-5p and piR-335-3p) and the remaining two miRNAs were classified both in Tables 1 and 3 (miR-137-3p and miR-455-3p).

### Validation of miRNAs involved in the reprogramming process

Individual qPCR assays were used to validate the results obtained in the miRNome analyses. A handful of miRNAs were selected from Tables 1, 2 and 3 and rat islets and exocrine tissue were used as controls.

**Fig 6:**
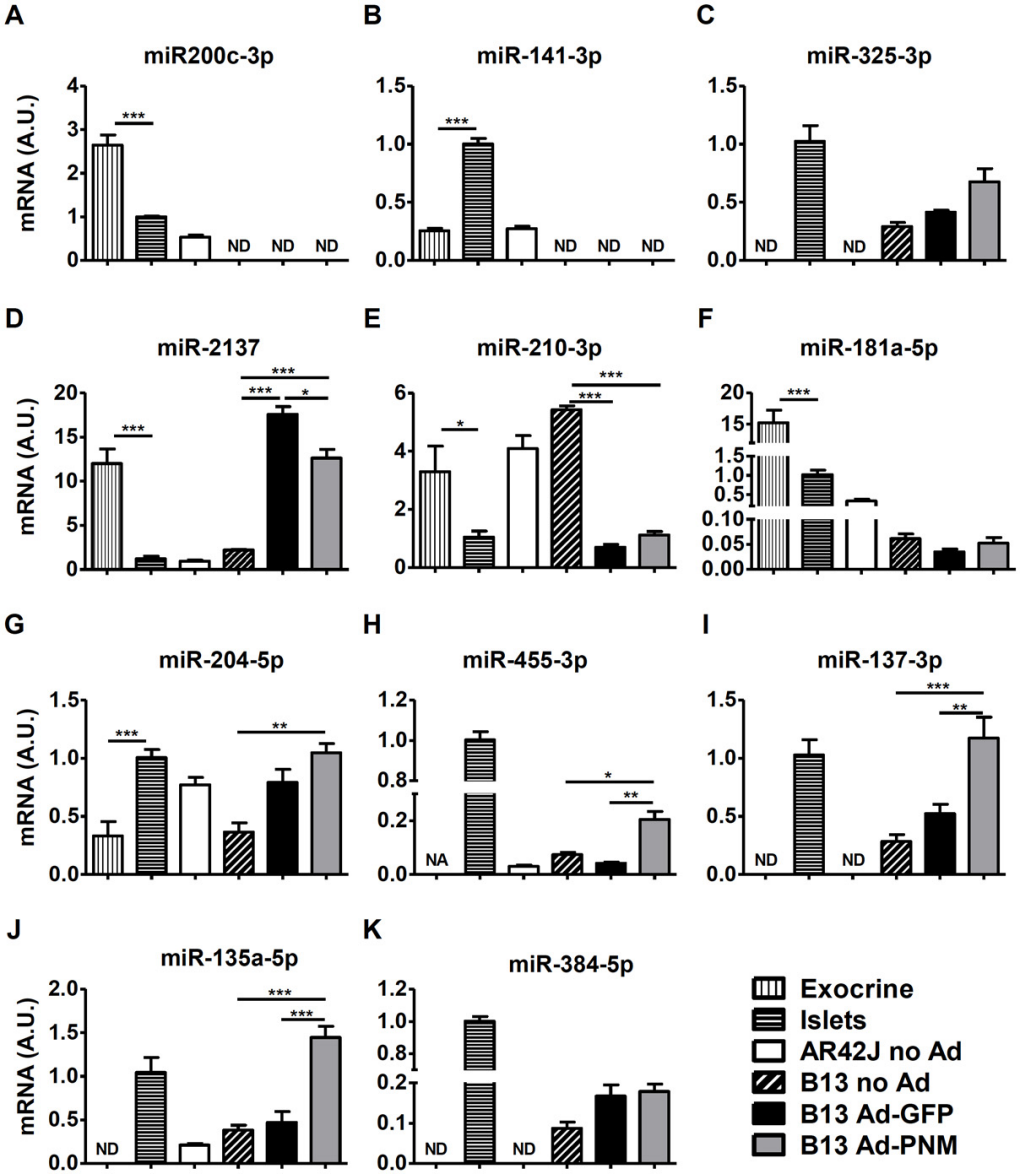
Expression levels of miRNAs of interest. Relative miRNA expression levels of miR-200c-3p (**A**), miR-141-3p (**B**), miR-325-3p (**C**), miR-2137 (**D**), miR-210-3p (**E**), miR-181a-5p (**F**), miR-204-5p (**G**), miR-455-3p (**H**), miR-137-3p (**I**), miR-135a-5p (**J**), miR-384-5p (**K**) in rat exocrine fractions, rat islets, AR42J cells, B13 cells and B13 transduced with Ad-GFP or Ad-PNM at 4 days post-transduction, quantified by individual qPCR assays. The results are depicted as means ± SEM. n = 4 for exocrine and islets controls, n = 3 for AR42J and B13 samples. ND, not detected. NA, not analyzed (because of 2 detected values and 2 non-detected values). *p < 0.05, **p<0.01, ***p<0.001, as determined by one-way ANOVA followed by a post hoc Tukey’s post test. A.U., arbitrary units.

The differential expression of miR-200c-3p, miR-141-3p and miR-325-3p between AR42J and non-transduced B13 cells (Table 1) was further confirmed by the individual qPCR assays (Fig 6A–6C).

Regarding changes in the expression profiles of miRNA mediated by adenoviral transduction, up-regulation of miR-2137 and miR-204-5p and down-regulation of miR-210-3p were also confirmed (Fig 6D, 6E and 6G), being the expression levels of miR-210-3p and miR-204-5p in Ad-transduced B13 cells similar to those displayed by rat islets. Conversely, miR-181a-5p expression levels in Ad-GFP transduced B13 cells were unchanged compared to non-transduced B13 cells (Fig 6F).

The up-regulation of miR-455-3p, miR-137-3p and miR-135a-5p due to PNM expression observed in our panels (Table 3) was also validated in this single PCR study (Fig 6H–6J). On the contrary, individual qPCR analysis did not reveal differences in the expression levels of miR-384-5p between the same groups (Fig 6K).

In summary, 11 candidates out of 69 (i.e. 15%) were selected for further confirmation using single qPCR analyses and the expression pattern was validated in 82% of the cases (9 out of 11), strongly suggesting that the vast majority of the results obtained by the miRNome screening were indeed reliable. Furthermore, expression levels of miR-137-3p, miR-135a-5p, miR-204-5p, miR-210-3p in reprogrammed B13 cells were consistent with the expression levels detected in rat islets and markedly differed from those displayed by rat exocrine tissue.

## Discussion

In the present work, the ability of the AR42J acinar cell line and its subclone B13 to be reprogrammed into insulin-producing cells after Ad-mediated simultaneous overexpression of the transcription factors *Pdx1*, *Ngn3* and *MafA* was investigated. Our data demonstrated that the B13 subclone could be more efficiently reprogrammed into a β cell phenotype than AR42J cells after adenoviral-mediated overexpression of PNM, and it was further confirmed that the B13 cell line is a suitable model for studying the mechanisms of acinar-to-β reprogramming, in agreement with previous reports [13,15–17]. However, it is worth mentioning that reprogrammed B13 cells were not fully differentiated β-cells as evidenced by the lack of glucose responsiveness *in vitro* (our data and [13]) and the low expression levels of endocrine markers compared to primary endocrine cells. It should be noted that the main purpose of our manuscript was not to generate fully differentiated β cells suitable for transplantation for the treatment of diabetes. Our purpose was to investigate which factors, in particular which miRNAs, are involved in the reprogramming process induced by PNM in exocrine cells.

It has been previously shown that adenoviral transduction can modulate reprogramming processes. Adenoviral vector-mediated expression of PNM in human bone-marrow derived mesenchymal stem cells (hMSCs) led to the activation of glucagon expression, whereas lentiviral vector-mediated expression of PNM in the same cells failed to induce the expression of endocrine hormones [29]. Wang and colleagues also showed that adeno-associated viral (AAV) vector-mediated overexpression of *Pdx1* and *Ngn3* did not modulate insulin production in hepatocytes of diabetic mice, whereas co-administration of these AAV vectors with an irrelevant adenoviral vector led to the correction of hyperglycemia [7]. Similarly, adenoviral-mediated delivery of PNM has been reported to reprogram acinar cells into β cells *in vivo* in immunodeficient mice [8,9], but AAV-mediated overexpression of PNM specifically in acinar cells of immunocompetent diabetic mice did not lead to similar reprogramming (unpublished data from the authors). Moreover, transgenic mice that were overexpressing PNM ubiquitously failed to convert acinar cells into insulin-producing cells [36]. In agreement with these observations, in this study, adenoviral transduction was shown to cause the downregulation of exocrine markers such as *Cpa1*, *Cela1* and *Ptf1a*, suggesting that adenoviral transduction facilitates reprogramming by dedifferentiating the exocrine phenotype of B13 cells.

Although *in vivo* PNM-mediated reprogramming of acinar cells is an interesting approach to treat T1D, to accomplish this, adenoviral transduction is necessary and animals must be immunosuppressed. Additionally, it has been shown that *in vivo* reprogrammed surrogate β cells in mice acquire functionality over time and need up to 7 months to exhibit full phenotypic maturity [8]. Moreover, human primary exocrine cells do not respond to adenoviral-mediated overexpression of *Pdx1*, *Ngn3*, *MafA* and chromatin-modifying agents and growth factors have been described to be required to permit the reprogramming into insulin producing cells [37]. Therefore, future efforts should be focused on improving the efficacy of the reprogramming protocol and on avoiding adenoviral transduction and immunosuppression. To this end, miRNAs are of special interest. For instance, the reprogramming of fibroblasts into iPSCs has been achieved merely by the overexpression of the miR302/367 cluster [38]. Likewise, a combination of miR-1, miR-133, miR-208 and miR-499 is capable of reprogramming fibroblasts into cardiomyocytes [21]. Regarding β cell reprogramming, co-transfection of miR-302 and PNM have been shown to induce the reprogramming of human hepatocytes into pancreatic progenitor cells [22], and lentiviral-mediated expression of miR-375 in iPSCs promoted the differentiation of these cells into insulin-producing islet-like clusters [23].

In this study we sought to identify which miRNAs are involved in acinar-to-β cell reprogramming. The analysis of differentially expressed miRNAs between AR42J and B13 cells by using miRNome panels revealed specific expression of all the members of the miR-200 family (miR-200a, miR-200b, miR-200c, miR-141-3p, miR-141-5p and miR-429) in the parental cell line AR42J, whereas none of these miRNAs were detected in the B13 subclone. There is strong evidence demonstrating that expression of the miR-200 family is enriched in terminally differentiated cells, such as epithelial tissues, and that it is undetected in pluripotent cells, such as mesenchymal cells [30–32]. Moreover, miR-200 family members target and inhibit ZEB1 and ZEB2 factors, which blocks epithelial-mesenchymal transition (EMT) and causes cells to maintain an epithelial state [33–35]. The EMT is a process by which epithelial cells lose their cell polarity and cell-cell adhesion while gaining migratory and invasive properties as they become mesenchymal stem cells: this results in multipotent cells that can differentiate into a variety of cell types [39]. A loss of E-cadherin expression is considered to be a fundamental event in the EMT, and many TFs can repress E-cadherin either directly or indirectly [40]. In B13 cells, the absence of the expression of all members of the miR-200 family correlated with higher expression levels of *Zeb1* and *Zeb2* and reduced expression of both *E-cad* mRNA and protein levels compared to its parental cell line. Moreover, a higher number of miRNAs with upregulated expression was found in AR42J *vs*. B13 cells. It has previously been described that the complexity of an miRNA expression profile is increased during differentiation in parallel with reduced expression levels of protein-coding genes [41]. Thus, a fully differentiated cell expresses a higher number of miRNAs than a pluripotent cell does [42]. The multipotency, or at least oligopotency, of B13 cells has also been demonstrated by the fact that these cells can be differentiated into hepatocytes [43]. Additionally, our results showed that B13 cells express lower levels of amylase and other exocrine markers compared to rat primary exocrine cells and the parental AR42J cells. Altogether, these data demonstrate that the B13 subclone is likely to exist in a less differentiated state than its parental cell line and as a consequence it presents a greater plasticity.

The miRNA expression profiles of Ad-GFP transduced B13 cells and non-transduced B13 cells obtained by miRNome panels were compared to further investigate the effects of adenoviral transduction. An upregulation of 7 miRNAs and a downregulation of 4 miRNAs were observed following Ad-GFP transduction. Among the overexpressed miRNAs, miR-148a, miR-335, miR-132 and miR-204 were identified, all of which have been reported to be overexpressed in β cells in comparison with α cells [44], suggesting that adenoviral transduction aids in cellular reprogramming toward a β cell lineage. In particular, miR-204 is one of the most highly–enriched miRs in β-cells (100-fold higher expression in β cells as opposed to α-cells) and has recently been shown to play an important role in the control of insulin production [45]. Notably, here we show that miR-204 expression levels in Ad-PNM reprogrammed B13 cells mirrored those of pancreatic islets.

To elucidate the effects of PNM overexpression, miRNA expression profiles of Ad-PNM and Ad-GFP transduced B13 cells were compared. In this case, upregulation of 6 miRNAs and downregulation of 2 miRNAs were found to be caused by PNM overexpression. Among the upregulated miRNAs, miR-134 and miR-22 were identified. These miRNAs have been previously reported to be upregulated in β cells compared to α cells [44]. Moreover, miR-134 represses several components of a network that controls pluripotency in its targeting of *Nanog*, *Oct4* and *Sox2* [46]. To the best of our knowledge, there are no reports describing a link between β cell development or function and the remaining overexpressed miRNAs that were identified in the present study: miR-455-3p, miR-384-5p, miR-137-3p and miR-135a-5p. Of note, using single qPCR assays we demonstrated that miR-455-3p, miR-137-3p and miR-135a-5p were expressed at very similar levels in reprogrammed B13 cells and pancreatic islets, suggesting a mechanistic relationship between these miRNAs and insulin production and/or β cell phenotype.

Thus, combinations of PNM and the miRNAs identified herein could potentially offer a more effective method for generating mature insulin-producing cells than current protocols.

With respect to clinical applications, the substitution of first generation adenoviral vectors with other type of vectors that are less immunogenic and toxic than adenovirus, such as AAV or helper-dependent adenoviral (HD-Ad) vectors, is desirable [47]. Our laboratory has shown that efficient and persistent transduction of the pancreas in vivo is possible using AAV and HD-Ad vectors via intraductal administration [48,49]. Thus, to induce acinar-to-β cell reprogramming in immunocompetent animals and humans, it might be feasible to co-express PNM and miRNAs of interest in vivo (for instance, those that are activated by first generation adenoviral vectors) using AAV or HD-Ad vectors. In the case of human acinar cells, direct *in vivo* reprogramming might be more advantageous compared with *in vitro* reprogramming of the same cells because human exocrine cells undergo dedifferentiation very quickly in culture forming a monolayer of mesenchymal cells [37].

Here, we demonstrate that B13 cells could be more efficiently reprogrammed into insulin-producing cells than could AR42J cells, which is probably related to their multipotent capacities. These cell lines presented a minimum of 59 miRNAs differentially expressed, and the plasticity of B13 cells could be explained, at least partially, by repression of the miRNA-200 family and E-cadherin as well as increased expression levels of Zeb1/Zeb2. We also demonstrated that adenoviral transduction induced dedifferentiation of acinar cells and that 11 miRNAs were putatively involved in this process, whereas 8 miRNAs were found to be associated with PNM expression. In summary, a list of 69 miRNAs putatively involved in acinar-to-β cell reprogramming was generated using miRNome panels (Tables 1, 2 and 3). To validate these observations, the expression of a selection of miRNAs was evaluated in rat pancreatic islets, rat exocrine tissue, ARJ42 and B13 (non-transduced, Ad-GFP- and Ad-PNM-transduced) cells by individual qPCR assays. Importantly, the expression pattern of more than 80% of the selected candidates was in agreement with the miRNome panels and the expression levels of miRNAs found in reprogrammed B13 cells were in many cases close to the expression levels displayed by islets.

Although further studies are warranted, the miRNAs identified herein might be of compelling importance for improving acinar to β cell reprogramming efficacy for the treatment of diabetes in the future.

## Supporting Information

S1 Fig Efficient transduction of AR42J and B13 cells with adenoviral vectors. Representative bright-field and fluorescence images are shown for AR42J (**A**) and B13 (**B**) cells that were either not transduced or transduced with either Ad-GFP or Ad-PNM vectors; images were taken at 2 days post-transduction. Green cells are GFP-producing cells. The transduction efficiency of the Ad-PNM vector was similar to that of the Ad-GFP control vector. In both cases, a vast majority of cultured cells were transduced with adenoviral vectors. Original magnification x200.
(PDF)

S2 Fig Relative expression levels of endocrine marker genes in reprogrammed B13 cells compared to primary rat endocrine cells (isolated pancreatic islets). Relative mRNA expression levels of rat endocrine markers *Ins1* (**A**), *Ins2* (**B**) *IAPP* (**C**) *NeuroD1* (**D**), and *Pax4* (**E**), insulin processing enzymes *Pcsk1* (**F**), *Pcsk2* (**G**) and *Cpe* (**H**) and the glucose transporter *Glut2* (**I**) in rat pancreatic islets and in B13 cells 4 days post-transduction with Ad-PNM. The results are depicted as means ± SEM. n = 4 for islets controls and n = 3 for B13 samples. *p < 0.05, **p < 0.01, ***p < 0.001, as determined by using Student’s *t* test. A.U., arbitrary units.
(PDF)

S3 Fig Relative expression levels of exocrine marker genes in B13 and ARJ42 cells compared to rat primary exocrine tissue. Relative mRNA expression of the exocrine markers *Amy* (**A**), *Cela1* (**B**), *Cpa1* (**C**) and *Ptf1a* (**D**) in rat exocrine fractions, AR42J and B13 cells. The results are depicted as means ± SEM. n = 4 for exocrine controls and n = 3 for AR42J and B13 samples. *p < 0.05, **p<0.01, ***p<0.001, as determined by one-way ANOVA followed by a post hoc Tukey’s post test. A.U., arbitrary units.
(PDF)

S4 Fig Downregulation of exocrine markers in B13 cells transduced with null adenoviral vectors. Relative mRNA expression of the exocrine markers *Cela1* (**A**), *Cpa1* (**B**), and *Ptf1a* (**C**) in B13 cells at 4 days after transduction with null adenoviral vectors (Ad-null). The results are depicted as means ± SEM. n = 3 wells per group. *p < 0.05, **p<0.01, ***p<0.001, as determined by one-way ANOVA followed by a post hoc Dunnett’s post test. A.U., arbitrary units.
(PDF)

S1 Table List of oligonucleotides pairs used for mRNA qPCR experiments. (PDF)

S2 Table miRNome panels raw data. (XLS)

S3 Table Ct values for differentially expressed miRNAs comparing AR42J cells to B13 cells. To minimize stochasticity observed at high Ct, values above 35 were considered non-detected (ND). 3 detected values versus ≥ 2 ND values were required to receive the label “Detected *vs*. ND”. NA, not analyzed (because of two Tm values). NV, no value. P values were determined by using Student’s *t* test. n = 3 wells per group.
(PDF)

S4 Table Ct values for differentially expressed miRNAs comparing B13 cells transduced with Ad-GFP to not transduced B13 cells. To minimize stochasticity observed at high Ct, values above 35 were considered non-detected (ND). 3 detected values versus ≥ 2 ND values were required to receive the label “Detected *vs*. ND”. P values were determined by using Student’s *t* test. n = 3 wells per group.
(PDF)

S5 Table Ct values for differentially expressed miRNAs comparing B13 cells transduced with Ad-PNM to B13 cells transduced with Ad-GFP. To minimize stochasticity observed at high Ct, values above 35 were considered non-detected (ND). 3 detected values versus ≥ 2 ND values were required to receive the label “Detected *vs*. ND”. P values were determined by using Student’s *t* test. n = 3 wells per group.
(PDF)
