# Supplementary figures and images for: Identification of miRNAs Involved in Reprogramming Acinar Cells into Insulin Producing Cells
Source: PLoS One. 2015 Dec 21;10(12):e0145116. doi: 10.1371/journal.pone.0145116 (PMC4686894; doi:10.1371/journal.pone.0145116)

S1 Figure

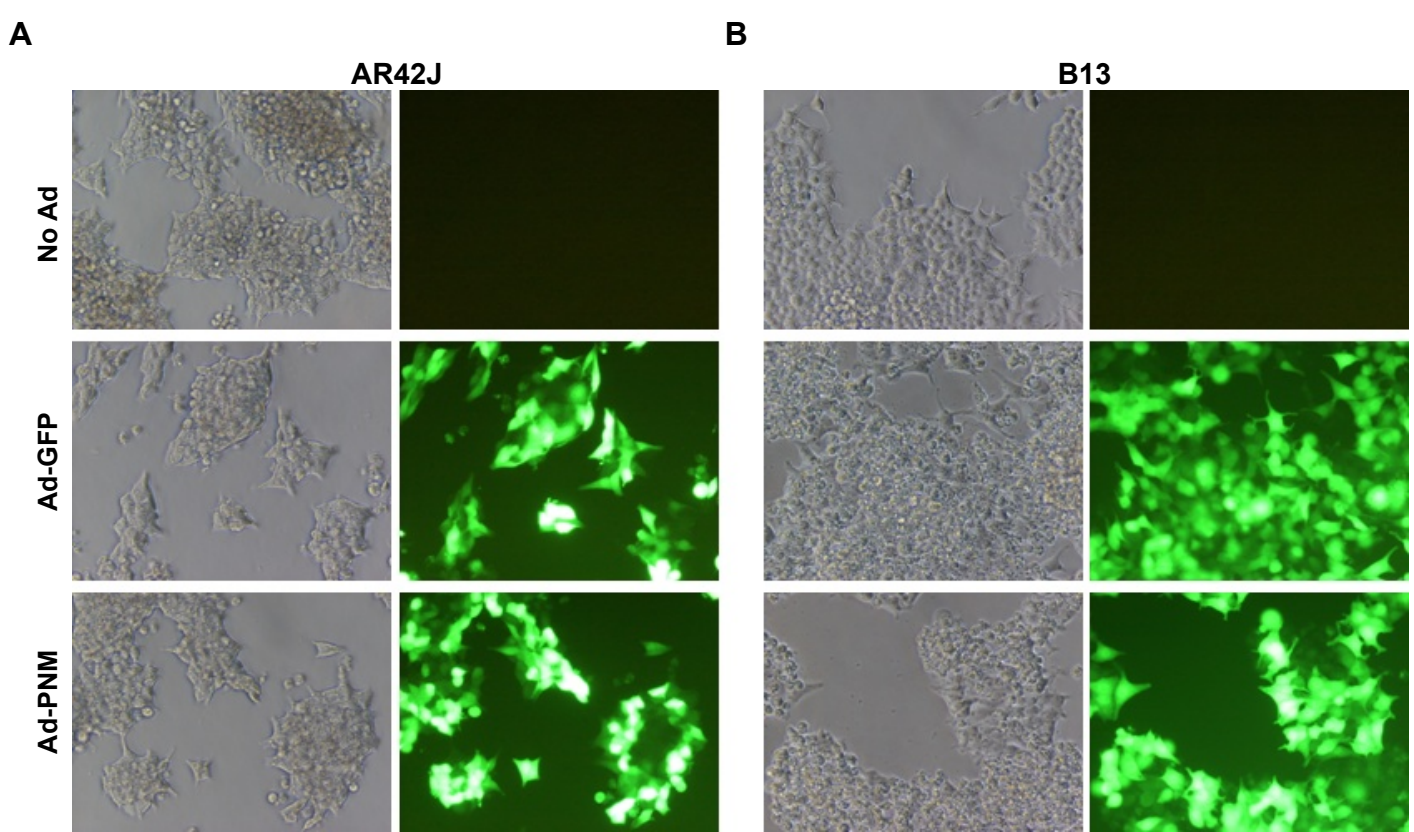

Supplement: S1 Fig — Representative bright-field and fluorescence images are shown for AR42J (A) and B13 (B) cells that were either not transduced or transduced with either Ad-GFP or Ad-PNM vectors; images were taken at 2 days post-transduction. Green cells are GFP-producing cells. The transduction efficiency of the Ad-PNM vector was similar to that of the Ad-GFP control vector. In both cases, a vast majority of cultured cells were transduced with adenoviral vectors. Original magnification x200. (PDF) [file pone.0145116.s001.pdf]

# S2 Figure

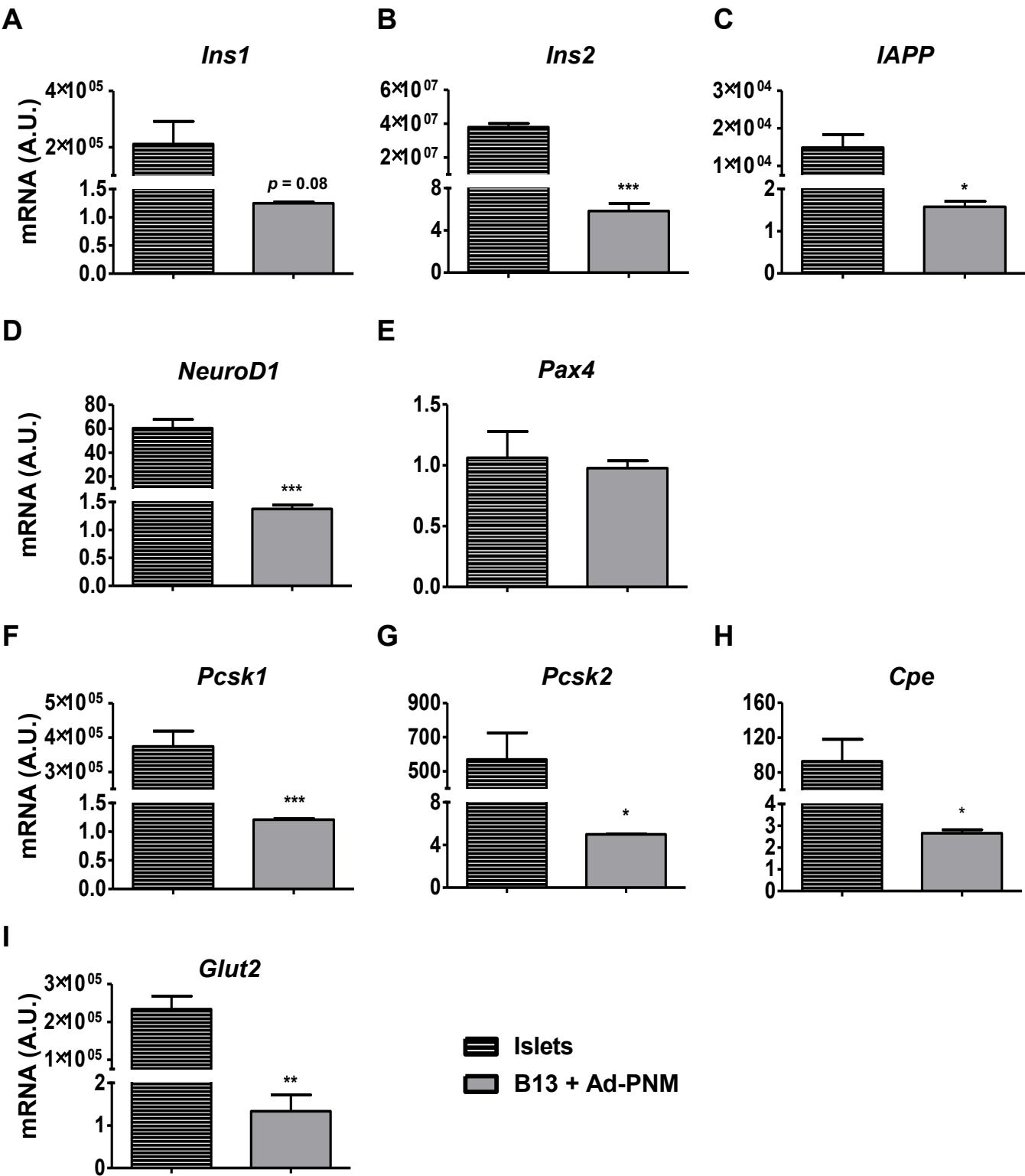

Supplement: S2 Fig — Relative mRNA expression levels of rat endocrine markers Ins1 (A), Ins2 (B) IAPP (C) NeuroD1 (D), and Pax4 (E), insulin processing enzymes Pcsk1 (F), Pcsk2 (G) and Cpe (H) and the glucose transporter Glut2 (I) in rat pancreatic islets and in B13 cells 4 days post-transduction with Ad-PNM. The results are depicted as means ± SEM. n = 4 for islets controls and n = 3 for B13 samples. *p < 0.05, **p < 0.01, ***p < 0.001, as determined by using Student’s t test. A.U., arbitrary units. (PDF) [file pone.0145116.s002.pdf]

S3 Figure

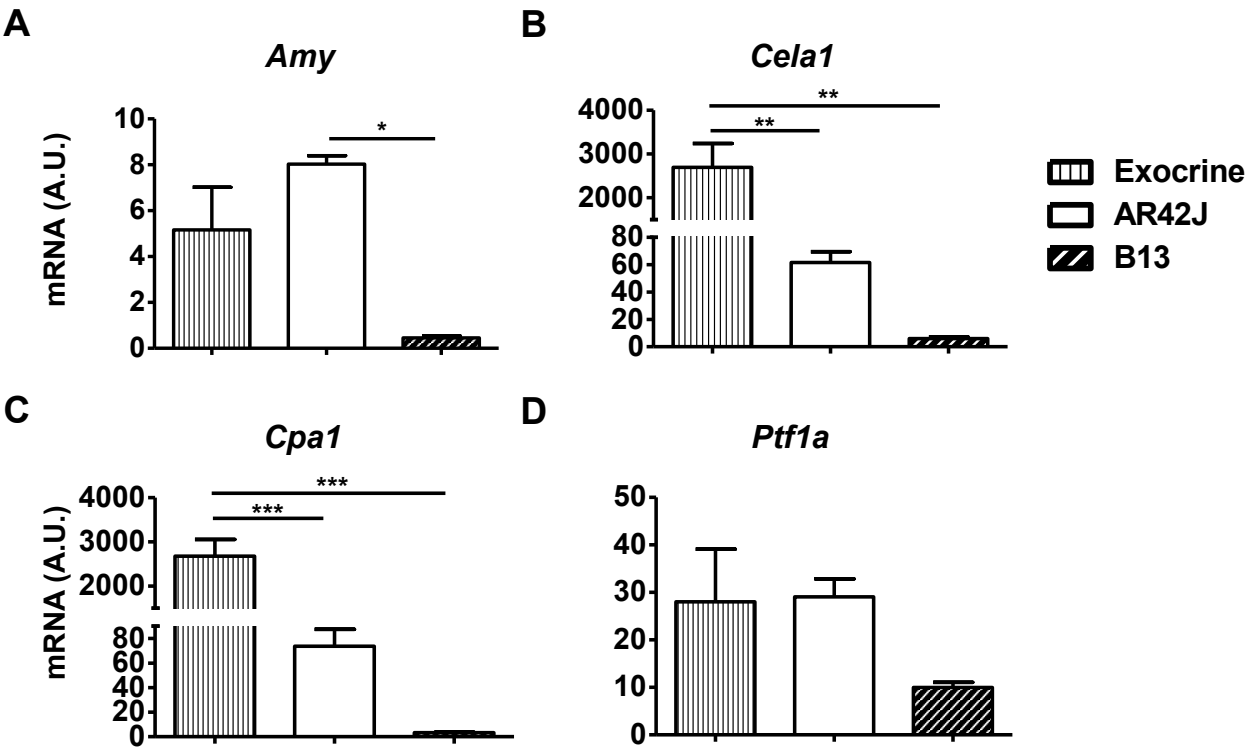

Supplement: S3 Fig — Relative mRNA expression of the exocrine markers Amy (A), Cela1 (B), Cpa1 (C) and Ptf1a (D) in rat exocrine fractions, AR42J and B13 cells. The results are depicted as means ± SEM. n = 4 for exocrine controls and n = 3 for AR42J and B13 samples. *p < 0.05, **p<0.01, ***p<0.001, as determined by one-way ANOVA followed by a post hoc Tukey’s post test. A.U., arbitrary units. (PDF) [file pone.0145116.s003.pdf]

# S4 Figure

A

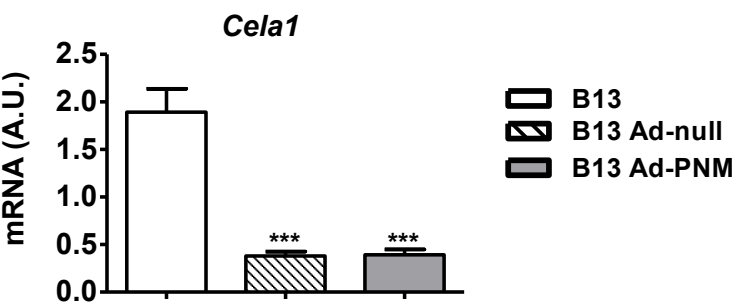

B

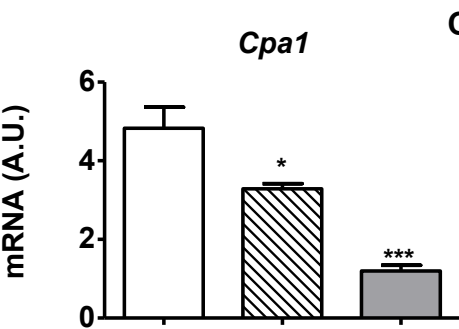

C

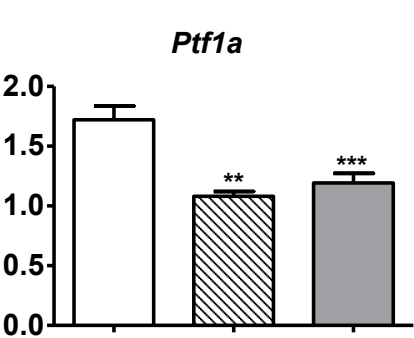

Supplement: S4 Fig — Relative mRNA expression of the exocrine markers Cela1 (A), Cpa1 (B), and Ptf1a (C) in B13 cells at 4 days after transduction with null adenoviral vectors (Ad-null). The results are depicted as means ± SEM. n = 3 wells per group. *p < 0.05, **p<0.01, ***p<0.001, as determined by one-way ANOVA followed by a post hoc Dunnett’s post test. A.U., arbitrary units. (PDF) [file pone.0145116.s004.pdf]
