# Supplementary material for: Identification of miRNAs Involved in Reprogramming Acinar Cells into Insulin Producing Cells
Source: PLoS One. 2015 Dec 21;10(12):e0145116. doi: 10.1371/journal.pone.0145116 (PMC4686894; doi:10.1371/journal.pone.0145116)
Supplement: S1 Table — (PDF) [file pone.0145116.s005.pdf]

S1 Table. List of oligonucleotides pairs used for mRNA qPCR experiments.

Gene	NCBI accession no.	Forward primer	Reverse primer
rnIns1	NM_019129.3	AATCATAGACCATCAGCAAGCA	CCACAAAGGTGCTGTTTGAC
rnIns2	NM_019130.2	TGTGGGGAGCGTGGATTCTT	AGTGCCAAGGTCTGAAGGTCA
rnIAPP	NM_012586.2	TGAGAGCTACACCTGTCGGA	ACATTGGTTGGTGGGAGGAC
rnNeuroD1	NM_019218.2	TCAACCCCCGGACTTTCTTG	GGGGACTGGTAGGAGTAGGG
rnPax4	NM_031799.1	TTTGTACCCAGGACAAGGCTC	GGAAGAGCTGGAGCCAAAAC
rnGlut2	NM_012879.2	GCCTGTGTATGCAACCATCG	ACGGCACAGAAAAACATGCC
rnPcsk1	NM_017091.2	ATCCTGTAGGCACCTGGACA	ACGAGGCTGCTTCATGTGTT
rnPcsk2	NM_012746.1	CTCTACGTTCAGCAACGGGA	CAAAGCCAATGCAAACACGC
rnCpe	NM_013128.1	CCGAGACCAAGGCTGTCATT	GTACCACTCCGCGTCTCATC
rnGck	NM_001270850.1	ACTGCCGAGATGATGAAGCAC	ACGATGTTGTTCCCTTCTGCT
rnAmylase	NM_031502.1	AGAGTAATGTCAAGTTACCGAAGGA	TGCCACAAGTAGTGTCTGGAT
rnCela1	NM_012552.3	TGAGCGTGCAGAAGATCGTG	TTGTTAGCCAGGATGGTTCCC
rnCpa1	NM_016998.3	TCCAGATCGGCAACACCTTT	AAACCAGACCCCACTAGCCT
rnPtf1a	NM_053964.1	TAACCAGGCCCAGAAGGTCAT	GAGCTGTTTTTCATCAGCCCAG
rnRplp0	NM_022402.2	GTTGAACATCTCCCCCTTCTCC	CAGTCGGGTAGCCAATCTGC
rnZeb1	NM_013164.1	GAGCCACCAGTGAAGGTGAT	GTCATTCTGGTCCTCCACGG
rnZeb2	NM_001033701.1	AAAAGCAGTTCCCTTCTGCGA	AGTGCTCGATAAGGTGGTGT
rnE-Cad	NM_031334.1	GCGAGAGCCAGACACATTCA	TCAGCCCGAGTGGAAATGAC
mmPdx1	NM_008814.3	GAAATCCACCAAAGCTCACG	CGGGTTCCGCTGTGTAAG
mmNgn3	NM_009719.6	CGCAAGAAGGCCAATGATCG	CCAGATGTAGTTGTGGGCGA
mmMafA	NM_194350.1	CTCCAGAGCCAGGTGGAG	GTACAGGTCCCGCTCCTTG

(rn, *rattus novergicus*; mm, *mus musculus*)
