# Supplementary material for: Identification of miRNAs Involved in Reprogramming Acinar Cells into Insulin Producing Cells
Source: PLoS One. 2015 Dec 21;10(12):e0145116. doi: 10.1371/journal.pone.0145116 (PMC4686894; doi:10.1371/journal.pone.0145116)
Supplement: S3 Table — To minimize stochasticity observed at high Ct, values above 35 were considered non-detected (ND). 3 detected values versus ≥ 2 ND values were required to receive the label “Detected vs. ND”. NA, not analyzed (because of two Tm values). NV, no value. P values were determined by using Student’s t test. n = 3 wells per group. (PDF) [file pone.0145116.s007.pdf]

S3 Table. Ct values for differentially expressed miRNAs comparing AR42J cells to B13 cells. To minimize stochasticity observed at high Ct, values above 35 were considered non-detected (ND). 3 detected values versus ≥ 2 ND values were required to receive the label "Detected *vs.* ND". NA, not analyzed (because of two Tm values). NV, no value. P values were determined by using Student's *t* test. n = 3 wells per group.

		AR42J no Ad			B13 no Ad				
	**ID sample**	**1**	**2**	**3**	**1**	**2**	**3**	**Ratio**	**P value**
**House Keeping**	miR-16-5p	23.7	23.9	23.9	24.1	23.5	23.7	-	-
**"AR42J no Ad" vs "B13 no Ad"**	miR-200c-3p	24.8	24.9	24.7	NV	NV	NV	Detected vs N.D.	-
	miR-141-3p	25.7	25.7	25.8	36.8	NV	NV	Detected vs N.D.	-
	miR-141-5p	28.8	28.7	28.8	NV	NV	NV	Detected vs N.D.	-
	miR-378a-3p	30.4	30.0	29.6	36.2	34.9	36.5	Detected vs N.D.	-
	miR-205-5p	30.2	30.7	30.5	NV	NV	NV	Detected vs N.D.	-
	miR-149-5p	30.7	30.5	31.4	NV	NV	NV	Detected vs N.D.	-
	miR-28-5p	31.1	31.8	31.1	NV	NV	NV	Detected vs N.D.	-
	miR-200b-3p	31.6	31.4	32.0	34.9	NV	NV	Detected vs N.D.	-
	miR-181b-5p	33.0	32.4	32.4	36.8	35.5	36.1	Detected vs N.D.	-
	miR-200c-5p	32.4	33.0	33.0	NV	NV	NV	Detected vs N.D.	-
	miR-429	33.0	32.9	32.5	NV	34.5	35.0	Detected vs N.D.	-
	miR-200a-3p	32.5	33.6	33.4	35.7	NV	37.8	Detected vs N.D.	-
	miR-483-3p	27.3	27.0	27.5	31.9	31.2	31.8	21.84	0.0004
	miR-28-3p	31.2	31.1	31.8	34.3	NA	34.8	8.48	0.0129
	miR-34a-5p	25.8	25.8	25.9	28.2	27.6	28.3	4.8	< 0.0001
	let-7i-5p	26.6	26.5	26.4	29.4	28.2	28.4	4.57	0.0007
	miR-181a-5p	30.0	30.1	30.4	32.6	31.4	32.5	4.21	0.0002
	let-7i-3p	32.4	32.8	32.7	34.9	33.9	NV	4.17	0.0004
	miR-192-5p	26.0	26.0	26.2	27.7	27.3	28.0	3.09	0.0004
	miR-30a-5p	27.0	26.8	27.0	28.8	28.0	28.2	2.89	0.0006
	miR-194-5p	27.1	27.3	27.1	29.1	28.2	28.5	2.86	0.0004
	miR-347	27.7	27.6	28.3	29.8	28.4	29.7	2.79	0.0152
	miR-122-5p	28.0	28.1	27.9	29.9	28.9	29.3	2.69	0.0012
	miR-374-5p	30.1	30.5	30.1	31.6	31.2	31.6	2.54	0.0038
	miR-30a-3p	28.2	28.6	28.8	30.3	29.3	29.7	2.47	0.0016
	let-7b-5p	24.6	24.8	24.4	26.4	25.2	25.7	2.42	0.0041
	let-7g-5p	24.7	24.7	24.6	26.2	25.4	25.8	2.34	0.0005
	miR-361-5p	29.4	28.9	29.1	30.5	30.1	30.1	2.32	0.0103
	miR-204-5p	27.4	27.3	27.1	28.6	28.1	28.4	2.32	0.0038
	miR-497-5p	26.6	26.5	26.9	28.1	27.4	27.8	2.22	0.0015
	let-7d-5p	26.5	26.4	26.2	27.8	27.2	27.2	2.18	0.0078

	miR-500-3p	32.9	33.5	34.0	34.3	34.4	34.8	2.13	0.0574
	miR-125b-5p	27.6	27.7	27.9	28.9	28.6	28.6	2.08	0.0001
	miR-181d-5p	31.4	31.1	31.2	32.9	31.9	31.8	2.06	0.0105
	miR-26b-5p	29.6	29.6	29.9	31.0	30.4	30.5	2.03	0.0007
	miR-421-3p	32.9	32.6	33.4	34.4	33.2	34.1	2.02	0.0511
**"B13 no Ad" vs "AR42J no Ad"**	miR-325-3p	34.7	35.1	36.6	29.1	28.3	28.9	Detected vs N.D.	-
	miR-325-5p	35.2	NV	NV	31.1	29.9	29.9	Detected vs N.D.	-
	miR-92b-3p	NV	NV	NV	33.8	32.3	32.9	Detected vs N.D.	-
	miR-142-3p	34.8	36.6	35.5	33.5	33.8	33.6	Detected vs N.D.	-
	miR-137-3p	38.8	38.1	38.7	34.5	33.5	33.7	Detected vs N.D.	-
	miR-27a-5p	36.5	35.0	35.1	34.3	33.9	34.4	Detected vs N.D.	-
	miR-330-3p	33.1	32.9	33.7	31.1	30.1	30.0	6.8	0.0039
	miR-101a-3p	30.0	30.0	30.4	28.4	27.8	28.2	3.75	< 0.0001
	miR-301a-5p	34.0	34.0	NV	32.9	32.1	32.5	3.55	0.0008
	miR-328a-3p	30.1	29.9	30.3	28.8	27.5	28.3	3.52	0.0082
	miR-301a-3p	29.0	29.1	29.0	27.6	26.8	27.2	3.31	< 0.0001
	miR-455-3p	31.0	30.9	31.1	30.2	28.9	29.0	3.07	0.0183
	miR-31a-3p	29.3	29.2	29.9	28.3	27.5	27.9	2.81	0.0003
	miR-193-5p	32.1	33.1	32.3	31.9	30.3	30.9	2.57	0.0339
	miR-2137	29.0	28.8	29.6	28.1	27.2	27.7	2.57	0.0012
	miR-1839-5p	30.9	30.6	30.4	29.9	28.5	29.2	2.55	0.0246
	miR-106b-5p	25.7	25.6	25.7	24.6	23.9	24.2	2.53	< 0.0001
	miR-7a-5p	27.0	27.0	26.8	26.6	25.2	25.3	2.3	0.038
	miR-153-3p	29.6	29.5	30.1	28.7	28.3	28.4	2.25	0.0016
	miR-365-3p	29.1	28.4	28.4	27.6	27.2	27.4	2.19	0.0033
	miR-25-3p	26.0	26.0	26.1	25.1	24.5	25.0	2.15	0.0003
	miR-29a-5p	29.9	30.0	29.9	29.2	28.3	29.0	2.09	0.0055
	miR-31a-5p	25.7	25.6	25.7	24.7	24.2	24.7	2.07	0.0012
