# Supplementary material for: Identification of miRNAs Involved in Reprogramming Acinar Cells into Insulin Producing Cells
Source: PLoS One. 2015 Dec 21;10(12):e0145116. doi: 10.1371/journal.pone.0145116 (PMC4686894; doi:10.1371/journal.pone.0145116)
Supplement: S4 Table — To minimize stochasticity observed at high Ct, values above 35 were considered non-detected (ND). 3 detected values versus ≥ 2 ND values were required to receive the label “Detected vs. ND”. P values were determined by using Student’s t test. n = 3 wells per group. (PDF) [file pone.0145116.s008.pdf]

S4 Table. Ct values for differentially expressed miRNAs comparing B13 cells transduced with Ad-GFP to not transduced B13 cells. To minimize stochasticity observed at high Ct, values above 35 were considered non-detected (ND). 3 detected values versus ≥ 2 ND values were required to receive the label "Detected *vs.* ND". P values were determined by using Student's *t* test. n = 3 wells per group.

		B13 no Ad			B13 + Ad-GFP				
	**ID sample**	**1**	**2**	**3**	**1**	**2**	**3**	**Ratio**	**P value**
**House Keeping**	miR-16-5p	24.1	23.5	23.7	24.5	23.9	23.6	-	-
**"B13 Ad-GFP" vs "B13 no Ad"**	miR-2137	28.1	27.2	27.7	25.0	24.7	24.5	9.1	0.0007
	miR-335-3p	27.9	27.1	27.5	26.4	26.1	25.6	3.23	0.0001
	miR-148a-5p	35.3	34.3	34.6	33.7	33.7	33.3	2.74	0.0151
	miR-421-3p	34.4	33.2	34.1	33.1	32.8	32.5	2.5	0.002
	miR-350	30.9	30.0	30.6	30.2	29.3	28.7	2.48	0.0066
	miR-204-5p	28.6	28.1	28.4	28.2	27.4	26.8	2.23	0.0041
	miR-132-3p	31.4	30.0	30.1	30.2	29.3	29.2	2.17	0.0088
**"B13 no Ad" vs "B13 Ad-GFP"**	miR-210-3p	27.0	26.4	26.6	31.0	30.1	29.1	8.71	< 0.0001
	miR-181a-5p	32.6	31.4	32.5	34.4	33.8	33.1	2.65	0.026
	miR-483-3p	31.9	31.2	31.8	33.6	33.3	32.4	2.36	0.003
	miR-181c-5p	31.6	31.0	31.2	33.5	32.8	31.6	2.1	0.0075
