# Supplementary material for: Identification of miRNAs Involved in Reprogramming Acinar Cells into Insulin Producing Cells
Source: PLoS One. 2015 Dec 21;10(12):e0145116. doi: 10.1371/journal.pone.0145116 (PMC4686894; doi:10.1371/journal.pone.0145116)
Supplement: S5 Table — To minimize stochasticity observed at high Ct, values above 35 were considered non-detected (ND). 3 detected values versus ≥ 2 ND values were required to receive the label “Detected vs. ND”. P values were determined by using Student’s t test. n = 3 wells per group. (PDF) [file pone.0145116.s009.pdf]

S5 Table. Ct values for differentially expressed miRNAs comparing B13 cells transduced with Ad-PNM to B13 cells transduced with Ad-GFP. To minimize stochasticity observed at high Ct, values above 35 were considered non-detected (ND). 3 detected values versus $\geq$ 2 ND values were required to receive the label “Detected *vs.* ND”. P values were determined by using Student’s *t* test. n = 3 wells per group.

		**B13 + Ad-GFP**			**B13 + Ad-PNM**				
	**ID sample**	**1**	**2**	**3**	**1**	**2**	**3**	**Ratio**	**P value**
**House Keeping**	miR-16-5p	24.5	23.9	23.6	24.0	24.3	23.5	**-**	**-**
**"B13 Ad-PNM" vs "B13 Ad-GFP"**	miR-134-5p	35.5	36.5	35.5	34.4	34.9	34.7	Detected vs N.D.	-
	miR-455-3p	31.0	30.3	29.8	28.4	28.5	28.3	3.78	0.0021
	miR-384-5p	33.2	31.7	31.4	30.9	30.7	29.6	3.17	0.0198
	miR-137-3p	34.3	33.2	32.4	31.2	32.3	31.6	2.88	0.0333
	miR-135a-5p	33.1	32.3	32.1	31.1	30.9	30.8	2.85	0.0126
	miR-22-5p	33.9	33.0	32.6	32.4	31.9	32.1	2.09	0.0892
**"B13 Ad-GFP" vs "B13 Ad-PNM"**	miR-335-3p	26.4	26.1	25.6	27.9	27.6	27.7	3.26	0.0006
	miR-148a-5p	33.7	33.7	33.3	34.2	35.0	34.3	2.24	0.038
